# The Effect of Body Mass Index on Melanoma Biology, Immunotherapy Efficacy, and Clinical Outcomes: A Narrative Review

**DOI:** 10.3390/ijms25126433

**Published:** 2024-06-11

**Authors:** Jente Jansen, Marjan Garmyn, Canan Güvenç

**Affiliations:** Department of Dermatology, University Hospitals Leuven, 3000 Leuven, Belgium; jente.jansen@student.kuleuven.be (J.J.); marjan.garmyn@uzleuven.be (M.G.)

**Keywords:** obesity paradox, body mass index, melanoma biology, immune checkpoint inhibitors, prognosis, survival

## Abstract

Recent studies indicate that a higher body mass index (BMI) might correlate with improved responses to melanoma treatment, especially with immune checkpoint inhibitors (ICIs), despite the general association of obesity with an increased risk of cancer and higher mortality rates. This review examines the paradoxical relationship between BMI and clinical outcomes in melanoma patients by exploring molecular links, the efficacy of immunotherapy, and patient survival outcomes. Our comprehensive literature search across the PubMed and Embase databases revealed a consistent pattern: increased BMI is associated with a better prognosis in melanoma patients undergoing ICI treatment. This “obesity paradox” might be explained by the metabolic and immunological changes in obesity, which could enhance the effectiveness of immunotherapy in treating melanoma. The findings highlight the complexity of the interactions between obesity and melanoma, suggesting that adipose tissue may modulate the immune response and treatment sensitivity favorably. Our review highlights the need for personalized treatment strategies that consider the metabolic profiles of patients and calls for further research to validate BMI as a prognostic factor in clinical settings. This nuanced approach to the obesity paradox in melanoma could significantly impact treatment planning and patient management.

## 1. Introduction

Cutaneous melanoma (CM), the most malignant form of skin cancer, arises from melanocytes, the pigment-producing cells in the skin. Its aggressive nature, characterized by rapid metastasis, underscores the necessity for early detection and intervention [1]. Despite advancements in understanding CM’s pathophysiology, the prognosis remains variable, influenced by a constellation of clinicopathologic features. These prognostic factors include Breslow thickness, mitotic rate, the presence of ulceration, age, sex, and anatomic location [1,2,3]. Intriguingly, recent studies have unveiled an apparent paradox: elevated body mass index (BMI) correlates with increased Breslow thickness, suggesting a dire prognosis [4,5,6,7]. Yet, paradoxically, it appears to enhance therapeutic responsiveness, a phenomenon not exclusive to CM but observed across various malignancies [8]. This “obesity paradox”, where obesity potentially exerts a protective effect, mitigating disease progression and elevating survival rates, leads to a deeper exploration into the complex interplay between adipose tissue and carcinogenesis [9,10,11].

**Figure 1:**
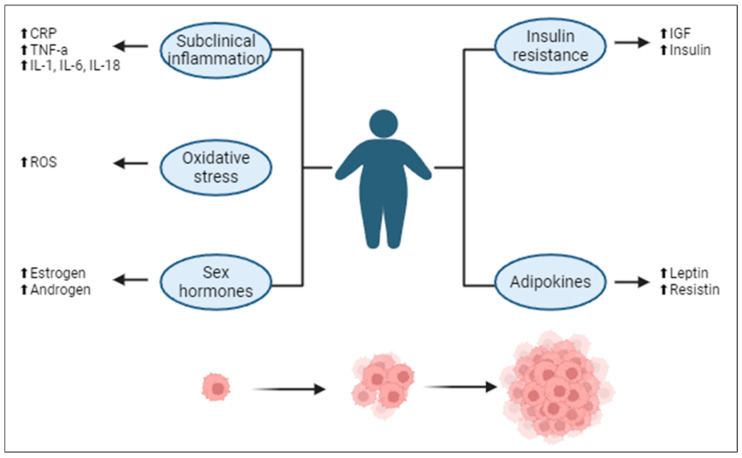
Hypotheses linking obesity and cancer. Abbreviations: CRP, c-reactive protein; TNF-α, tumor necrosis factor alpha; IL-1, interleukin-1; IL-6, interleukin-6; IL-18, interleukin-18; ROS, reactive oxygen species; IGF-1, insulin-like growth factor-1; FASN, fatty acid synthase; Cav-1, caveolin-1. Arrows: ⬆, increase; ➡, results in. Adapted from Talib et al. [13]. Created with Biorender.

Hypotheses propose that the excess adipose tissue in obesity serves as a reservoir for fat-soluble antioxidants, mitigating oxidative stress and, by extension, CM progression [12,13] (Figure 1). Moreover, the altered adipokine secretion profile in obese individuals might modulate the immune response favorably against CM [13,14]. Obesity is also closely associated with insulin resistance and chronic inflammation, which contribute to cancer progression. Elevated levels of inflammatory cytokines such as IL-6, TNF-α, and CRP are common in obese individuals and have been linked to tumor growth and metastasis [12,13,14]. Additionally, our prior observations emphasize the role of augmented estrogen receptor (ER) pathways in non-metastasizing thick CM among the elderly, attributed to the possible contribution of adipose tissue to estrogen production post-menopause, further influencing immune responses [15,16].

BMI, a ratio of weight to height, stands as the predominant metric for assessing obesity, endorsed by the World Health Organization with defined thresholds: obesity refers to BMI ≥ 30 kg/m^2^, overweight to BMI 25–29.9 kg/m^2^, normal weight to BMI 18.5–24.9 kg/m^2^, and underweight to BMI < 18.5 kg/m^2^. While it offers a standardized population-level estimate of body composition, it is not flawless since it does not consider other factors, such as muscle mass, bone density, and fat distribution. Nevertheless, its utility as a surrogate marker for adiposity, associated with increased risk of chronic conditions including cardiovascular diseases and gastrointestinal and hormone-sensitive organ cancers, remains undisputed [7,12,17,18].

This comprehensive review aims to appraise BMI’s influence on CM prognosis, highlighting the underlying biological mechanisms. We aim to study the effects of BMI on melanoma biology, immunotherapy efficacy, and clinical outcomes to offer a comprehensive understanding of its relationship with melanoma progression, treatment response, and patient outcomes.

## 2. Results

**Figure 2:**
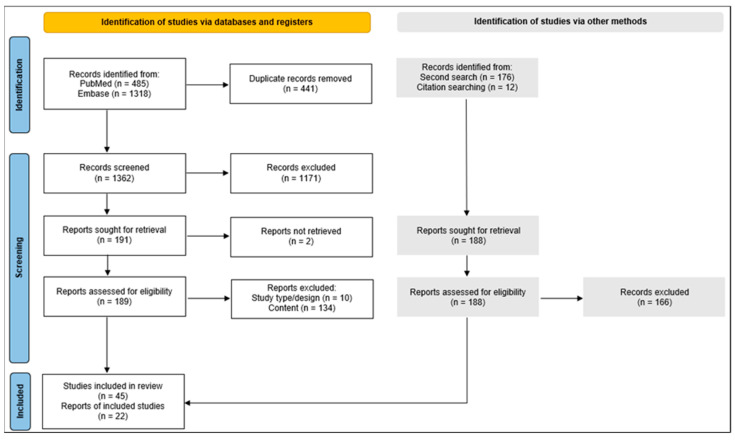
PRISMA flow diagram. On 11 May 2023, a systematic database search yielded 1803 articles. Post-duplicate removal, 1362 articles were screened based on title and abstract using predefined in- and exclusion criteria; this screening excluded 1171 articles: 21 for language discrepancies, 123 due to publication type, and 1027 for content relevance. Subsequent full-text evaluation led to the exclusion of an additional 144 articles. A second search (grey boxes) identified 176 studies, and a thorough examination of all reference lists added 12 more studies. In total, 67 articles were included in this narrative review.

Sixty-seven studies were included in this narrative review (Figure 2).

### 2.1. Obesity and Melanoma Biology

#### 2.1.1. Obesity-Induced Pathophysiological States and Immune Dysfunction

Olszanska et al. (2021) explored the impact of the melanoma microenvironment, particularly focusing on adipose tissue and its secretions, such as adipokines, which play a major role in modulating melanoma cell activities [19]. Increases in leptin and resistin encourage the growth and spread of melanoma cells and also alter the tumor microenvironment (TME) [20,21]. Kulkarni et al. (2021) and Molinelli et al. (2023) expanded on the topic by discussing the inflammatory and metabolic disturbances caused by obesity, emphasizing the dysregulation of adipose tissue and the emission of inflammatory and tumor-promoting adipokines [22,23]. Talib et al. (2023) highlighted chronic inflammation, insulin resistance, and hormonal imbalances as key factors through which obesity promotes carcinogenesis [13] (Figure 1).

Furthermore, obesity was linked to impaired immune system functions, affecting the behavior of dendritic cells, T cells, natural killer cells, and macrophages [24]. Wang et al. (2018) and Roccuzzo et al. (2023) showed that obesity-induced immune changes, particularly the dysfunction and increased amount of programmed cell death protein 1 (PD-1)-mediated T cells, have been shown to improve the effectiveness of immunotherapies. This suggests that obesity may induce a form of tumorigenic immune dysfunction that can be targeted with ICIs such as PD-1/PD-L1 inhibitors [21,24]. In addition, Incorvaia et al. (2023) investigated the response to immunotherapy, finding that pretreatment with low levels of soluble immune checkpoints accompanied by increased tumor-infiltrating lymphocytes can predict better outcomes in immunotherapy [25]. On the other hand, some patients with CM showed no response to ICIs [6,26]. Khojandi et al. (2021) identified alternative pathways, such as oxidative stress and lipid metabolism, as mediators in the intricate connection between obesity and resistance to immunotherapy in melanoma, with oxidized low-density lipoproteins playing a pivotal role in supporting tumor cell survival. This mechanism was facilitated by heme-oxygenase-1, which shielded tumor cells from apoptosis [27].

Grabacka et al. (2020) studied the molecular interactions between metabolic processes and melanoma development in obese patients, focusing on the role of peroxisome proliferator-activated receptor alpha (PPARα) and its influence on the mammalian target of rapamycin (mTOR) signaling pathway. They highlighted how PPARα activation and subsequent mTOR signaling contribute to metabolic reprogramming in melanoma [28]. Moreover, they highlighted the significance of reactive oxygen species (ROS) and the potential benefits of pharmacological strategies aimed at modulating the PPAR and AMP-activated protein kinase (AMPK) pathways. The use of AMPK activators, such as phenformin, emerged as a promising avenue to improve the effectiveness of ICIs in melanoma by targeting myeloid-derived suppressor cells. This strategy illustrates the broader potential of metabolic interventions in augmenting immunotherapy for melanoma, even for tumor subtypes resistant to conventional treatments like nivolumab or ipilimumab [28]. A schematic overview of the key findings from the included articles is listed in Table 1.

#### 2.1.2. Adipocyte-Rich Tumor Microenvironment Alters Melanoma Metabolism

Zheng et al. (2023) studied the expression patterns of Acyl-CoA synthetase medium-chain 3 (ACSM3) in melanoma, uncovering links with obesity and patient survival that emphasize the genetic foundations of the obesity paradox in this cancer. ACSM3, involved in butyric acid metabolism and regulated by the androgen receptor (AR), showed reduced expression in both melanoma and normal skin tissues. Notably, ACSM3 and AR expressions were positively correlated in obese males and overweight females, with ACSM3 expression also positively associated with BMI in these groups. Interestingly, elevated ACSM3 levels were linked to better overall survival in males, but not in females, suggesting that obesity-related metabolic changes, such as those involving short-chain FAs like butyric acid, could play a significant role in melanoma progression and survival outcomes [29]. Zhu et al. (2023) added to our comprehension of the TME in CM by identifying triggering receptor expression on myeloid cells 2 (TREM2) as a prognostic marker. TREM2 overexpression, observed in obese melanoma patients, has been associated with enhanced immune cell infiltration and activation, potentially through the modulation of the TME to favor anti-tumor immune responses. This study highlights the advantageous role of high-level immune infiltration, including CD8+ T cells, neutrophils, B cells, and dendritic cells, in extending the cumulative survival rate of CM patients, suggesting a direct link between TREM2 expression and the activation of immune response mechanisms within the TME [30].

Moreover, Smith et al. (2020) examined the effects of external factors, like a fat-rich diet, on melanoma cell behavior, emphasizing how obesity-induced metabolic disruptions, such as elevated serum insulin, insulin-like growth factor 1 (IGF-1), and adipokine production, create a pro-tumorigenic environment. Interestingly, this condition not only accelerates melanoma growth but also increases tumor responsiveness to targeted treatments, revealing a complex interplay between obesity-related metabolic changes and melanoma dynamics [14]. Hahn et al. (2022) further investigated this metabolic phenotype associated with obesity and its impact on treatment responses in metastatic melanoma, noting that obese patients experienced better outcomes with ICIs and targeted therapies. Thus, the metabolic alterations accompanying obesity, such as increased levels of FAs and adipokines, might make tumors more receptive to such interventions [34]. Assumpção et al. (2022) focused on the broader implications of obesity in cancer, emphasizing the obesity paradox observed in immunotherapy responses. They discussed how obesity-induced alterations in the immune system, and its crosstalk with cancer cells can paradoxically improve outcomes in cancer immunotherapy [12].

**Figure 3:**
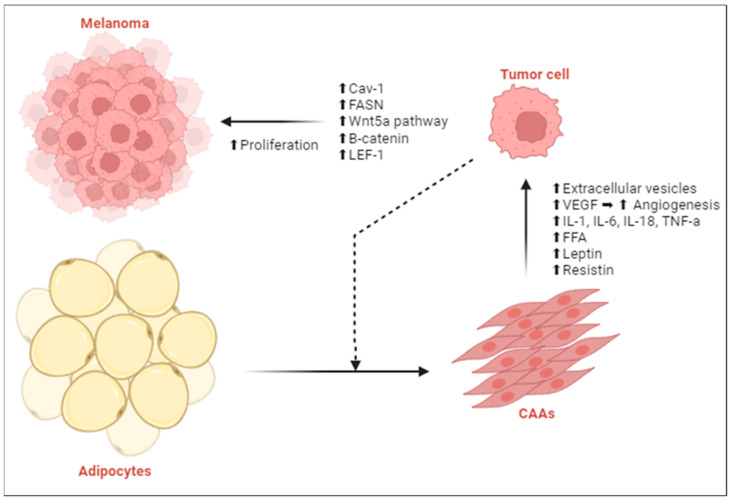
Tumor-adipose tissue crosstalk. In the tumor microenvironment (TME), adipocytes are converted into cancer-associated adipocytes (CAAs). This transformation involves delipidation, resulting in smaller, fibroblast-like cells. These altered adipocytes exhibit dysregulated secretion of various molecules, thereby enhancing melanoma progression. Abbreviations: IL-1, interleukin-1; IL-6, interleukin 6; IL-18, interleukin 18; TNF-α, tumor necrosis factor alpha; FFA, free fatty acid; FASN, fatty acid synthase; Cav-1, caveolin-1; VEGF, vascular endothelial growth factor; LEF-1, lymphoid enhancer-binding factor 1. Arrows: ⬆, increase; ➡, results in; dashed line, stimulatory modification.Adapted from Olszanska et al. [19]. Created with Biorender.

Robado de Lope et al. (2017), Zoico et al. (2017), and Moraes et al. (2021) investigated the influence of the TME on melanoma metastasis and progression, focusing on the role of adipocytes and extracellular vesicles on tumor behavior. Their studies show that tumor cells induce changes in adjacent adipocytes, leading to delipidation and transformation into cancer-associated adipocytes (CAA). These CAAs release free FAs, enhancing fatty acid metabolism and elevating the expression of chemoattractants in tumor cells, thereby boosting their malignancy. Their research highlights the important function of the TME in creating a more aggressive melanoma phenotype [31,32,33]. Clement et al. expanded on this topic, providing an in-depth analysis of how obesity leads to a more favorable microenvironment for melanoma growth and disease progression. They identified multiple mechanisms by which obesity contributes to melanogenesis, including an increase in Breslow thickness, augmented tumor growth in situ due to cytokines released from hypertrophic adipose tissue, and the effect of CAAs on promoting melanoma invasiveness and metastasis. The release of FAs through lipolysis, adipocyte-driven remodeling of the extracellular matrix to support angiogenesis and invasion, and the creation of a cytokine-rich environment that promotes tumor growth were pinpointed as crucial connections between obesity and the progression of melanoma [35] (Figure 3). A schematic overview of the key findings from the included articles is listed in Table 1.

### 2.2. BMI and Melanoma Prognosis

The majority of the currently available evidence was from research on the link between BMI and the efficacy of melanoma treatments, particularly focusing on ICIs. Studies have consistently demonstrated a positive association between a higher BMI and improved outcomes, including overall survival (OS) and progression-free survival (PFS), in melanoma patients receiving ICIs [9,10,36]. Further subgroup analysis revealed that a BMI > 25 kg/m^2^ was a reliable cut-off for determining significantly better OS [37]. This pattern was observed across various malignancies, not exclusive to CM, suggesting a strong connection between adiposity and the success of immunotherapy [8]. Nie et al. (2021) further explored this dynamic, indicating that while there is a general trend of better survival outcomes with increased BMI, the benefits do not significantly differ between overweight and obese patients, illustrating a plateau effect to the survival advantage above a BMI > 30 kg/m^2^. The elevated risk associated with a higher BMI may also contribute to increased mortality from non-cancer-related conditions, such as cardiovascular and cerebrovascular diseases, which could diminish the benefits derived from ICIs [11]. In addition, Cortellini et al. (2020), Bastacky et al. (2021), and Harrell Shreckengost et al. (2021) revealed that patients with increased BMI benefited from better survival outcomes but also faced an increased incidence of immune-related adverse events (irAEs) when treated with PD-1/PD-L1 inhibitors [7,38,39].

Research by Nitipir et al. (2020) found that weight gain during treatment, indicative of an increasing BMI, correlated with improved PFS, highlighting BMI’s predictive value during treatment [40]. Similarly, Kondo et al. (2018) and Cortellini et al. (2019) emphasized BMI’s prognostic importance in melanoma and other malignancies treated with ICIs [41,42]. Conversely, Rutkowski et al. (2020), Deckers et al. (2021), and Zepeda-Najar et al. (2021) presented varied outcomes regarding obesity’s prognostic significance in melanoma [6,26,43]. Di Filippo et al. (2021), Lee et al. (2022), and Antoun et al. (2023) nuanced this discussion by suggesting that BMI alone may not fully capture the impact of a patient’s body composition on prognosis, highlighting the importance of considering other factors such as skeletal muscle mass [44,45,46]. A schematic overview of the key findings from the included articles is listed in Table 2.

**Figure 4:**
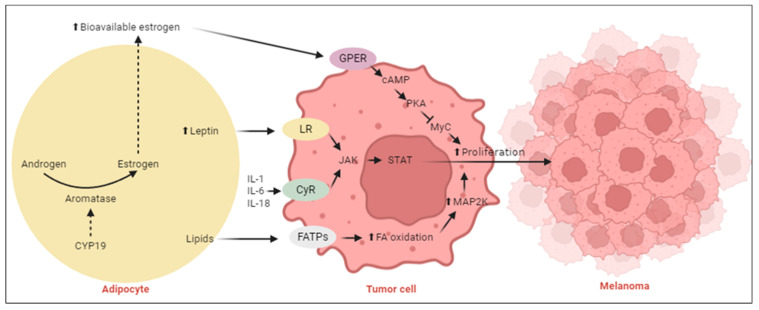
Interplay between obesity, sex hormone metabolism, inflammation, and melanoma progression. The enzyme aromatase converts androgens into estrogens, consequently activating the G protein-coupled estrogen receptor (GPER) signaling pathway. Activation of this pathway has been shown to suppress melanoma cell proliferation and enhance the efficacy of immune checkpoint inhibitors (ICI). Abbreviations: CYP19, cytochrome P450 family 19; GPER, G protein-coupled estrogen receptor; LR, leptin receptor; FATPs, fatty acid transporter proteins; FA, fatty acid; CyR, cytokine receptor; JAK, Janus kinase; STAT, signal transducer and activator of transcription; cAMP, cyclic adenosine 3′,5′-monophophate; MyC, MyC proto-oncogene; MAP2K, mitogen-activated protein kinase kinase; IL-1, interleukin 1; IL-6, interleukin-6; IL-18, interleukin-18; TNF-α, tumor necrosis factor alpha. Arrows: ⬆, increase; ➡, stimulates; dashed line, influences; ⊣, inhibits. Adapted from Roccuzzo et al. [21]. Created with Biorender.

Additionally, McQuade et al. (2018) noted a gender-specific survival benefit for obese men receiving targeted and immune therapies [47]. Concurrently, epidemiological data show that men experience higher melanoma incidence and mortality rates compared to women [1,48,49,50,51,52]. These observations suggest that gender-specific biological mechanisms could be shaping both the development of CM and its response to treatments. These gender-based differences may be due to the interaction between fat and sex hormone metabolism, potentially through the differential expression profiles of AR and ER [21] (Figure 4).

## 3. Discussion

Obesity presents a paradoxical effect in the context of melanoma. On one hand, excess body fat promotes the proliferation of melanoma cells, yet on the other, it appears to render the cancer more amenable to immunotherapy. This has led researchers to speculate about the metabolic pathways that might underlie the observed beneficial correlation between higher BMI and enhanced responsiveness to ICI therapy. Such findings suggest that a higher BMI could serve as a predictive marker for favorable treatment outcomes in melanoma patients [53].

### 3.1. Melanoma Biology

Our findings from the literature review emphasize the duality of obesity in melanoma progression and treatment response [14,35]. On the one hand, obesity promotes the proliferation of melanoma cells and may hinder the efficacy of traditional treatments, such as chemotherapy, through the action of obesity-associated adipokines like leptin and resistin. These substances not only accelerate melanoma cell growth but also modify the TME, creating conditions that favor tumor growth and resistance to therapy [19,20,21,22,23]. On the other hand, the altered immune landscape associated with obesity, characterized by impaired function and increased counts of dendritic cells and T cells, paradoxically seems to enhance the effectiveness of immunotherapies [12]. This includes agents targeting PD-1, suggesting that obesity-induced immune dysfunction might be exploitable in enhancing responses to ICIs [21,24,25,27]. The interplay between metabolic dysregulation and immune modulation forms a critical axis in understanding the obesity paradox in melanoma. Our findings from various studies underline chronic inflammation, insulin resistance, and the alteration of systemic and local hormone levels as major mechanisms through which obesity exacerbates melanoma progression [13,34]. Simultaneously, these conditions may render the melanoma more amenable to ICIs, possibly due to the modulation of the TME and immune evasion tactics employed by tumor cells [31,32,33,35]. Additionally, the expression of specific genes like ACSM3 appears to influence melanoma survival, hinting at genetic factors that underlie the observed clinical outcomes [29]. Moreover, the presence of factors such as TREM2 within the TME has been associated with favorable patient outcomes [30].

Integrating these findings, it becomes clear that obesity exerts a multi-dimensional influence on melanoma. The paradoxical nature of obesity’s impact on both promoting tumor progression and enhancing the efficacy of ICIs presents a complex challenge. This challenge necessitates a deeper understanding of the underlying mechanisms and interactions at play, which could unveil novel therapeutic targets and strategies for managing melanoma, thus underscoring the critical need for a holistic approach in researching and treating melanoma, taking into account the pervasive influence of obesity on this malignancy’s metabolism, growth, and response to therapy.

### 3.2. Melanoma Prognosis

Our analysis extends the discussion on the obesity paradox by highlighting the improved treatment outcomes in melanoma patients with higher BMI [9,10]. The data from various studies suggest that obesity may serve as a predictive marker for better responses to immunotherapy [36,37]. This finding is particularly relevant in the context of emerging evidence suggesting that elevated BMI is associated with better OS and PFS among patients undergoing ICI therapy [40,41]. These outcomes highlight a potential shift in how patient nutritional and metabolic status could be considered in tailoring melanoma treatment strategies. Moreover, recent studies identified a higher occurrence of irAEs in overweight and obese patients, which, interestingly, is associated with more favorable treatment outcomes [7,38,39]. This connection implies that irAEs, serving as indicators of the effectiveness of immunotherapy, occur more frequently in individuals with a higher BMI, suggesting a stronger antitumor response.

Nevertheless, the dynamics between BMI, treatment efficacy, and prognosis are complex. Variations in how BMI affects treatment outcomes indicate that the survival benefits associated with higher BMI might differ based on gender, type of treatment, and other variables [6,26,43,44,45,46,47,51,52]. Moreover, the counterintuitive nature of these results, where factors typically linked to negative health outcomes emerge as indicators of better survival and response to treatment, calls for a reassessment of BMI’s role in cancer metabolism and immunotherapy responses [11].

### 3.3. Confounding Variables: “Explaining the Inconsistencies”

The identification of confounding variables is necessary to understand the conflicting results of obesity on the efficacy of ICIs in melanoma treatment. The current evidence identifies several (modifiable) host factors, such as age, sex, microbiome composition, gender, vitamin D level, dietary patterns, and sarcopenia, which affect CM prognosis [47,48,51,52,54,55]. The discrepancies seen in obesity’s effects on ICI efficacy stem from these confounders, collectively shaping melanoma prognosis. Targeting these modifiable factors may unveil opportunities to boost immunotherapy effectiveness in melanoma through customized treatment plans acknowledging individual patient characteristics. This approach advocates for an encompassing strategy in melanoma care, prioritizing patient stratification not just by genetic and tumor-specific criteria but also by (modifiable) host factors. Advancements in this research domain could pave the way for more effective and personalized therapeutic interventions.

#### 3.3.1. Age-Related Variability and Gut Microbiome

The processes of aging are intricately linked due to a reduced functional capacity of the immune system, a condition termed immunosenescence. This deterioration holds profound implications for the success of immunotherapies, which are based on the ability of the immune system to identify and kill cancer cells. The variability in therapeutic response to ICIs highlights the impact of age on treatment outcomes, with a cut-off at 65 years to distinguish between older and younger cohorts [56].

Additionally, the aging process is correlated with changes in the composition of the gut microbiome, characterized by gut dysbiosis. These dysbiotic shifts within the intestinal flora enhance proinflammatory innate immune responses. The interaction between the microbiome and immune response after immunotherapy suggests that individual variances in microbiome composition may impact the effectiveness of these treatments [56]. Moreover, studies indicate that individuals with obesity exhibit microbiome configurations that align with enhanced responses to ICI [57,58]. This underscores the microbiome’s significant role in shaping immune responses, presenting the manipulation of microbiota composition as a viable therapeutic avenue [54,56].

#### 3.3.2. Sex-Based Differences

Research highlights gender-specific discrepancies in melanoma outcomes, particularly the enhanced survival benefit observed in obese men receiving targeted and immune therapies [47]. The connection between fat metabolism and sex hormone dynamics provides a viable explanation: variations in the expression profiles of AR and ER might influence both disease progression and therapeutic responsiveness [21]. This notion is supported by the documented disparities in melanoma incidence rates across genders, underscoring the possible impact of hormonal dynamics [1,51,52]. Further research is necessary to study these mechanisms and assess the potential of targeting sex hormone pathways in the management of melanoma [15,29].

#### 3.3.3. Vitamin D, Sunlight, and Diet

The contributions of serum vitamin D and dietary habits to melanoma prognosis and therapeutic outcomes have been thoroughly investigated. Associations between serum vitamin D, sun exposure, and risk of melanoma suggest a protective effect of vitamin D, particularly in individuals with a normal to elevated BMI [59,60,61]. Dietary factors, like the consumption of meat, fish, and fat rich in omega-3 and omega-6, correlate with Breslow thickness at diagnosis, where a healthier diet is inversely related to melanoma risk [54,62]. These findings emphasize diet and vitamin D as modifiable factors that might affect melanoma dynamics in the context of ICI therapy [63].

#### 3.3.4. Skeletal Muscle Mass and Sarcopenia

Sarcopenia, defined as the degradation of skeletal muscle mass, is identified as a major determinant of survival outcomes in melanoma patients. Associations between lower serum creatinine, a surrogate marker for skeletal muscle mass, and diminished survival outcomes moderate the observed gender-based obesity paradox [48]. Research investigating sarcopenia as a prognostic variable in melanoma patients suggests its relevance for ICI treatment responses, underlining the necessity to evaluate body composition beyond BMI in assessing melanoma prognosis and treatment success [55,64,65].

Moreover, it is important to recognize that poor nutritional status and malnutrition can lead to immune system exhaustion. Malnutrition may impair immune function, reducing the efficacy of immunotherapies and increasing the risk of irAEs. Thus, the nutritional status of patients should be carefully monitored and managed to optimize treatment outcomes [65].

### 3.4. Clinical Implications: “When Fat Becomes Favorable”

The link between BMI and CM presents a complex clinical challenge, informed by a body of research that intersects the fields of oncology, molecular biology, and immunology. The body of evidence describes BMI as a significant determinant not just of melanoma risk and progression, but also of the treatment response and survival rates. We studied the biological mechanisms of how BMI might be intertwined with melanoma outcomes, proposing BMI’s potential as a prognostic factor in melanoma care. These biological insights, alongside clinical findings, support the notion of BMI as a viable indicator to predict melanoma outcomes. BMI’s role could extend to guiding clinical trial design and individualizing treatment strategies, by leveraging it as a patient stratification criterion. For instance, acknowledging the superior performance of certain immunotherapies in patients with obesity could guide clinicians toward therapies best suited to a patient’s BMI category. Early identification of individuals with a predisposition towards adverse outcomes, based on BMI, might trigger preventive measures like dietary or lifestyle adjustments to diminish obesity-related complications. Additionally, tracking BMI fluctuations during therapy could offer valuable clues regarding the treatment’s impact and the patient’s health trajectory. Embedding BMI into the melanoma treatment framework advocates for an integrative patient care strategy, one that considers the tumor’s nature as well as the patient’s metabolic and immune profile, aiming to elevate patient care and potentially boost survival rates.

### 3.5. Strengths and Limitations

Our review highlights the significant role of BMI in shaping the course and effectiveness of melanoma treatments, presenting itself as a crucial resource for healthcare professionals aiming to customize treatment plans according to the specific needs of their patients. We focus attention on the gaps and inconsistencies in the current data, positioning this review as a catalyst for further research into the nuanced relationship between BMI and melanoma outcomes. We encourage a more in-depth exploration of how BMI interacts with the disease process and treatment response.

Acknowledging the inherent limitations of literature reviews, it is possible that studies may have been overlooked or that new relevant research emerged after our literature search (publication bias). To minimize the risk of overlooking significant studies, a comprehensive search strategy was developed in conjunction with experienced biomedical reference librarians. Additionally, the reference lists of included research papers were meticulously examined to capture any studies potentially omitted in the initial search. A second search was done to incorporate any recent publications.

### 3.6. Future Directions

The intricate relationship between BMI and CM outcomes, as revealed by the available evidence, highlights an area for further study. The distinct effects of BMI on the risk, advancement, and treatment response of melanoma call for an in-depth, more refined investigation of these dynamics. Given the heterogeneous nature of melanoma, encompassing various subtypes with unique genetic characteristics, behaviors, and treatment responses, a meta-analysis that performs subgroup analysis by tumor type could shed light on BMI-related prognostic factors specific to each subtype. This could lead to more precisely targeted treatment approaches.

In addition, subgroup analyses based on Tumor, Node, Metastasis (TNM) Classification could identify stage-specific effects of BMI, uncovering opportunities for intervention or unique prognostic indicators at different points in the disease’s progression.

Moreover, the variable influence of BMI on the effectiveness of melanoma treatments, from surgical interventions to targeted and immunotherapies, warrants thorough investigation. Analyzing subgroups by treatment type could reveal how BMI affects the success of each therapeutic option, facilitating the development of tailored treatment strategies.

A significant limitation in the current literature is the reliance on static BMI measurements, without considering the trajectory of BMI changes over time. This simplification overlooks the potential impact of progressive wasting or weight gain before and during therapy, which could have significant implications for treatment outcomes and immune function. Future studies should incorporate longitudinal analyses of nutritional status to better understand these dynamics.

Future research efforts should aim to incorporate diverse populations to affirm the applicability of their findings across different genetic backgrounds, environmental conditions, and healthcare systems, which could all affect the BMI–melanoma outcome relationship.

## 4. Materials and Methods

This narrative review was conducted adhering to the Preferred Reporting Items for Systematic Reviews and Meta-Analyses (PRISMA) guidelines [66].

A detailed and comprehensive search strategy was developed by merging specific keywords relevant to “cutaneous melanoma”, “body mass index”, and “adipose tissue”. The search strings, constructed with precise Boolean operators, were employed to query PubMed and Embase databases on 11 May 2023. The exact search strings used, including the combinations of keywords and operators, are outlined in Appendix A. The selection of PubMed and Embase was based on their extensive coverage of biomedical literature, ensuring a broad and inclusive retrieval of relevant studies.

A single investigator conducted the preliminary screening of potential articles, initially based on their titles and abstracts, followed by a thorough full-text analysis. Eligibility for inclusion was restricted to studies published in the most recent five-year period leading up to the final search cut-off date of 11 May 2023. An additional search was executed on 22 October 2023, to incorporate any recent publications. The inclusion criteria were carefully selected to cover a range of study designs including systematic reviews, meta-analyses, randomized controlled trials, cohort studies, and cross-sectional studies, specifically those reporting on predetermined outcomes associated with BMI, melanoma biology, and clinical outcomes. Exclusion criteria were applied to case reports or series, conference abstracts, letters to editors, and editorials, with the literature review being confined to works published in either English or Dutch. The reference lists of included studies from both the initial and subsequent search were further checked for relevance. This process is illustrated in Figure 2.

The search results were imported into Endnote (Version 20, Clearview Analytics, Philadelphia, PA, USA), where duplicates were identified and removed. The unique articles were transferred into Rayyan for rigorous screening, based on the established in- and exclusion criteria, as outlined in Appendix B [67]. Data extraction was performed using a standardized form to ensure consistency and comprehensiveness, capturing relevant study characteristics, participant demographics, and outcomes of interest. This process was completed by one reviewer

## 5. Conclusions

Our review highlights the interaction between BMI and melanoma outcomes, mediated by complex biological mechanisms that influence both tumor biology and treatment response. The clinical community strives toward more personalized melanoma treatment, the incorporation of BMI as a clinical predictor could enhance patient stratification, guide therapeutic decision-making, and encompass a more holistic approach to patient care. Future research should aim to further elucidate the mechanisms linking obesity and melanoma prognosis and validate BMI as a reliable predictor for treatment outcomes, paving the way for its integration into clinical practice.

## Figures and Tables

**Table 1:** Obesity and melanoma biology. Abbreviations: LDL, low-density lipoproteins; PPARα, peroxisome proliferator-activated receptor alpha; ACMS3, acyl-CoA synthetase medium-chain 3; TREM2, triggering receptor expression on myeloid cells 2; CAAs, cancer-associated adipocytes; Cav-1, caveolin-1; β-cat, beta-catenin; LEF-1, lymphoid enhancer-binding factor 1; FAs, fatty acids. Arrow: ⬆, increase.

Reference	Obesity	Melanoma Biology
Olszanska [19] Malvi [20] Molinelli [23]	Adipokine dysregulation	⬆ Inflammation ⬆ Immune dysfunction ⬆ Tumor growth ⬆ Melanoma proliferation
Kulkarni [22]	Macrophage polarization	⬆ Melanoma proliferation
Talib [13] Smith [14] Assumpção [12]	Chronic inflammation	⬆ Insulin resistance ⬆ Adipokine dysregulation ⬆ Tumor growth ⬆ Melanoma proliferation
Wang [24] Roccuzzo [21] Incorvaia [25]	T cell dysfunction	⬆ Efficacy of immunotherapy
Khojandi [27]	Oxidized LDL	⬆ T cell suppression ⬆ Melanoma’s immune escape
Grabacka [28]	PPARα upregulation	⬆ Prognosis
Zheng [29]	ACSM3 upregulation	
Zhu [30]	TREM2 upregulation	
Zoico [31]	⬆ CAAs	⬆ Activation of Cav-1, Wnt5a, β-cat, LEF-1 ⬆ Tumor growth ⬆ Melanoma proliferation
Robado de Lope [32] Moraes [33]	⬆ Extracellular vesicles	⬆ Pro-inflammatory proteins and FAs ⬆ Tumor growth ⬆ Melanoma proliferation

**Table 2:** BMI and melanoma prognosis. Abbreviations: BMI, body mass index; OS, overall survival; PFS, progression-free survival; irAEs, immune-related adverse events. Arrow: ➡, results in; ⬆, increase.

Reference	BMI Cohorts	Melanoma Prognosis
Petrelli [9]	BMI ≥ 30 kg/m^2^ BMI < 30 kg/m^2^	BMI ≥ 30 kg/m^2^ ➡ improved survival (OS)
You [36]	BMI ≥ 25 kg/m^2^ BMI < 25 kg/m^2^	BMI ≥ 25 kg/m^2^ ➡ improved survival (OS, PFS), irAEs ⬆
Cortellini [38]		
Bastacky [39]		
An [10]		BMI ≥ 25 kg/m^2^ ➡ improved survival (OS, PFS)
Nie [11]		
Zepeda-Najar [6]		
Antoun [46]		
McQuade [47]		
Rutkowski [26]		No survival advantage
Deckers [43]		
Di Filippo [44]		
Lee [45]		

## Abbreviations

ACMS3	Acyl-CoA synthetase medium-chain 3
AMPK	AMP-activated protein kinase
AR	Androgen receptor
BMI	Body mass index
CAA	Cancer-associated adipocytes
Cav-1	Caveolin-1
CM	Cutaneous melanoma
CRP	C-reactive protein
ER	Estrogen receptor
FAs	Fatty acids
FASN	Fatty acid synthase
FFA	Free fatty acid
ICI	Immune checkpoint inhibitor
IGF-1	Insulin-like growth factor-1
IL-1	Interleukin-1
IL-6	Interleukin-6
IL-18	Interleukin-18
irAEs	Immune-related adverse events
LEF-1	Lymphoid enhancer-binding factor 1
LDL	Low-density lipoproteins
mTOR	Mammalian target of rapamycin
OS	Overall survival
PD-1	Programmed cell death protein 1
PD-L1	Programmed death-ligand 1
PFS	Progression-free survival
PPARα	Peroxisome proliferator-activated receptor alpha
PRISMA	Preferred Reporting Items for Systematic Reviews and Meta-Analyses
ROS	Reactive oxygen species
TME	Tumor microenvironment
TNF-α	Tumor necrosis factor alpha
TNM	Tumor, Node, Metastasis
TREM2	Triggering receptor expressed on myeloid cells 2
VEGF	Vascular endothelial growth factor
Wnt5a	Wnt family member 5A
β-cat	Beta-catenin

## Appendix A

**Table A1:** Search Strings in PubMed and Embase. Abbreviation: *, truncation.

**Database**	**Search String**
PubMed	(“Melanoma”[Mesh] OR “melanoma*”[tiab] OR “melanocarcinoma* ”[tiab] OR “melano-carcinoma*”[tiab] OR “Melanoma, Cutaneous Malignant”[Supplementary Concept] OR “melanomalignoma*”[tiab] OR “naevocarcinoma*”[tiab] OR “nevocarcinoma*”[tiab] AND (“Body Mass Index”[Mesh] OR “body mass”[tiab] OR “quetelet*”[tiab] OR “BMI”[tiab] OR “thinness”[Mesh] OR “thinness”[tiab] OR “leanness”[tiab] OR “underweight”[tiab] OR “ideal body weight”[Mesh] OR “body weight”[tiab] OR “healthy weight”[tiab] OR “overweight”[Mesh] OR “overweight”[tiab] OR “obesity”[Mesh] OR “obes*”[tiab] OR “Adipose Tissue”[Mesh] OR “adipose tissue”[tiab] OR “fatty tissue”[tiab] OR “fat tissue”[tiab] OR “body fat”[tiab] OR “fat deposition”[tiab] OR “Body Fat Distribution”[Mesh] OR “body fat distribution”[tiab] OR “adiposity”[tiab] OR “beige fat”[tiab] OR “white fat”[tiab] OR “abdominal fat”[tiab] OR “subcutaneous fat”[tiab] OR “brown fat”[tiab] OR “Skinfold Thickness”[Mesh] OR “skinfold thickness*”[tiab] OR “skin fold thickness*”[tiab] OR “skin fold measurement*”[tiab ] OR “skinfold measurement*”[tiab])) NOT “Uveal melanoma”[Supplementary Concept] OR “uveal-melanoma*”[tiab] OR “eye-melanoma*”[tiab] OR “Mucous Membrane”[Mesh] OR “mucous-membrane*”[tiab] OR “mucosa”[tiab] OR “central nervous system melanoma*”[tiab].
Embase	((‘Melanoma’/exp OR ‘melanoma*’:ti,ab,kw OR ‘melanocarcinoma*’:ti,ab,kw OR ‘melano-carcinoma*’:ti,ab,kw OR ‘melanomalignoma*’:ti,ab,kw OR ‘cutaneous melanoma’/exp OR ‘naevocarcinoma*’:ti,ab,kw OR ‘nevocarcinoma*’:ti,ab,kw AND (‘Body mass’/exp OR ‘body mass’:ti,ab,kw OR ‘BMI’:ti,ab,kw OR ‘quetelet* index’:ti,ab,kw OR ‘BMI chart’/exp OR ‘BMI’:ti,ab,kw OR ‘underweight’/exp OR ‘underweight’:ti,ab,kw OR ‘thinness’:ti,ab,kw OR ‘leanness’:ti,ab,kw OR ‘ideal body weight’/exp OR ‘body weight’:ti,ab,kw OR ‘healthy weight’:ti,ab,kw OR ‘obesity’/exp OR ‘obes*’:ti,ab,kw OR ‘overweight’:ti,ab,kw OR ‘Adipose tissue’/de OR ‘adipose tissue’:ti,ab,kw OR ‘fatty tissue’:ti,ab,kw OR ‘fat tissue’:ti,ab,kw OR ‘body fat’/exp OR ‘body fat’:ti,ab,kw OR ‘fat deposition’:ti,ab,kw OR ‘body fat distribution’/exp OR ‘body fat distribution’:ti,ab,kw OR ‘adiposity’:ti,ab,kw OR ‘beige adipose tissue’/exp OR ‘beige fat’:ti,ab,kw OR ‘white adipose tissue’/exp OR ‘white fat’:ti,ab,kw OR ‘abdominal fat’/exp OR ‘abdominal fat’:ti,ab,kw OR ‘subcutaneous fat’/exp OR ‘subcutaneous fat’:ti,ab,kw OR ‘brown adipose tissue’/exp OR ‘brown fat’:ti,ab,kw OR ‘skinfold thickness’/exp OR ‘skinfold thickness*’:ti,ab,kw OR ‘skin fold thickness*’:ti,ab,kw OR ‘skin fold measurement*’:ti,ab,kw OR ‘skinfold measurement*’:ti,ab,kw)) NOT ‘Eye melanoma’/exp OR ‘eye melanoma*’:ti,ab,kw OR ‘uveal melanoma*’:ti,ab,kw OR ‘mucosa’/exp OR ‘mucosa’:ti,ab,kw OR ‘mucous membrane*’:ti,ab,kw OR ‘central nervous system melanoma’/exp OR ‘central nervous system melanoma*’:ti,ab,kw) NOT (‘conference abstract’:it OR ‘editorial’:it OR ‘letter’:it).

## Appendix B

**Table A2:** In- and Exclusion Criteria.

Inclusion criteria	Language: research articles written in English or Dutch Study type/design: original research articles, systematic reviews published in the last five years (interval 2018–2023) Content: In vivo studies using human specimens or lab animals, e.g., rodents Only studies examining melanoma Full-text availability (open access or via KU Leuven Association)
Exclusion criteria	Language: research articles not written in English or Dutch Study design: case reports or series, conference abstracts, letters to editors, and editorials Content: In vitro studies, e.g., cell cultures Studies investigating other tumor types No full-text availability
